# Infection, pathology and interferon treatment of the SARS-CoV-2 Omicron BA.1 variant in juvenile, adult and aged Syrian hamsters

**DOI:** 10.1038/s41423-022-00923-9

**Published:** 2022-10-18

**Authors:** Lunzhi Yuan, Huachen Zhu, Peiwen Chen, Ming Zhou, Jian Ma, Xuan Liu, Kun Wu, Rirong Chen, Qiwei Liu, Huan Yu, Lifeng Li, Jia Wang, Yali Zhang, Shengxiang Ge, Quan Yuan, Qiyi Tang, Tong Cheng, Yi Guan, Ningshao Xia

**Affiliations:** 1 State Key Laboratory of Molecular Vaccinology and Molecular Diagnostics, National Institute of Diagnostics and Vaccine Development in Infectious Diseases, School of Life Sciences, School of Public Health, Xiamen University, Xiamen, Fujian China; 2 State Key Laboratory of Emerging Infectious Diseases, School of Public Health, Li Ka Shing Faculty of Medicine, The University of Hong Kong, Hong Kong SAR, China; 3 Guangdong-Hong Kong Joint Laboratory of Emerging Infectious Diseases/Joint Laboratory for International Collaboration in Virology and Emerging Infectious Diseases, Joint Institute of Virology (STU/HKU), Shantou University, Shantou, Guangdong China; 4 EKIH Pathogen Research Institute, Futian District, Shenzhen, Guangdong China; 5 Department of Microbiology, Howard University College of Medicine, Washington, DC USA

**Keywords:** SARS-CoV-2 Omicron, Lung pathology, Innate immune response, Interferon treatment, Infection, Mechanisms of disease

## Abstract

The new predominant circulating SARS-CoV-2 variant, Omicron, can robustly escape current vaccines and neutralizing antibodies. Although Omicron has been reported to have milder replication and disease manifestations than some earlier variants, its pathogenicity in different age groups has not been well elucidated. Here, we report that the SARS-CoV-2 Omicron BA.1 sublineage causes elevated infection and lung pathogenesis in juvenile and aged hamsters, with more body weight loss, respiratory tract viral burden, and lung injury in these hamsters than in adult hamsters. Juvenile hamsters show a reduced interferon response against Omicron BA.1 infection, whereas aged hamsters show excessive proinflammatory cytokine expression, delayed viral clearance, and aggravated lung injury. Early inhaled IFN-α2b treatment suppresses Omicron BA.1 infection and lung pathogenesis in juvenile and adult hamsters. Overall, the data suggest that the diverse patterns of the innate immune response affect the disease outcomes of Omicron BA.1 infection in different age groups.

## Introduction

The severe acute respiratory syndrome coronavirus 2 (SARS-CoV-2) Omicron variant, the newly identified variant of concern (VOC) [1], has now become predominant across the world. It possesses an unusually large number of mutations in the spike protein, which constitute the molecular basis of its exceptional transmissibility and immune escape capacity [2, 3]. Indeed, Omicron infects fully vaccinated populations [4–7] and escapes the majority of existing SARS-CoV-2 neutralizing antibody therapies [8–10]. In the early outbreaks of Omicron, most patients exhibited mild symptoms and rapid recovery [6, 11]. Recently, the hospitalization rate and severity have dramatically increased [12, 13], particularly in cohorts such as very young children [14, 15]. As many features of Omicron remain unclear, understanding its infection efficiency and pathogenicity in different age groups is critical and fundamental for evidence-based intervention and treatment measures. Although Omicron has been demonstrated to exhibit reduced viral replication and cause body weight loss in several adult rodent models [16, 17], systematic pathological analyses are still lacking. Moreover, the patterns of virus-host interplay upon Omicron infection have yet to be clarified. Whether juveniles or aged individuals show infection outcomes similar to those of adults after Omicron infection is unknown. Here, we used the Syrian hamster, a sensitive rodent model for SARS-CoV-2, to explore the viral replication, pathogenicity, innate immune response, and treatment of Omicron in different age groups.

## Results

**Fig. 1:**
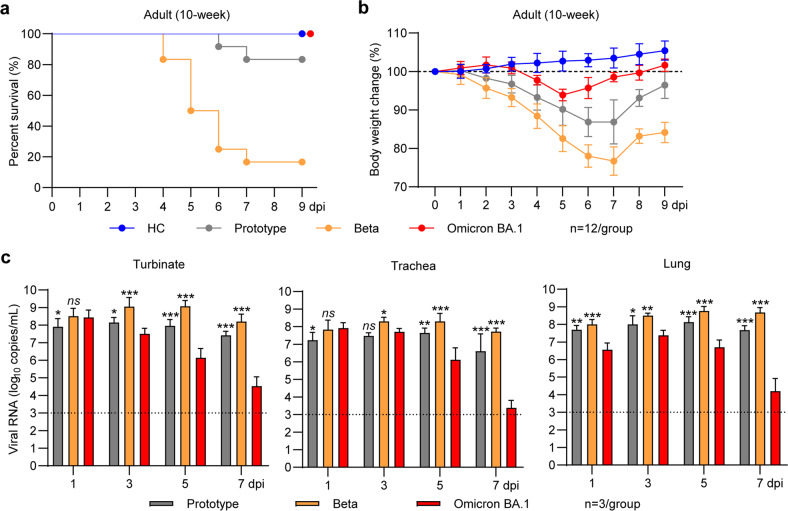
Pathogenicity and infectivity of the SARS-CoV-2 prototype strain and the Beta and Omicron BA.1 variants in adult hamsters. **a** Survival rates and **b** body weight loss of SARS-CoV-2-infected male hamsters and healthy controls (HCs) from 0 to 9 dpi (*n* = 12). In another parallel experiment, three hamsters were euthanized at 1, 3, 5, and 7 dpi. **c** The viral RNA loads in respiratory tract organs, including the nasal turbinates, trachea, and lungs, were measured by RT‒PCR (*n* = 3). Significance was calculated using two-way ANOVA (*P < 0.05; **P < 0.01; ***P < 0.001; ns nonsignificant)

We first compared the pathogenicity and replication efficiency of the SARS-CoV-2 Omicron BA.1 sublineage with the prototype virus and the highly pathogenic Beta variant. Adult Syrian hamsters (10 weeks old, *n* = 12/group) were inoculated by the intranasal route with 1 × 10^5^ PFU of SARS-CoV-2 prototype, Beta and Omicron BA.1 strains as previously described [18, 19]. From 0 to 9 days post-infection (dpi), all of the Omicron BA.1-infected hamsters survived, while two prototype- and 10 Beta-infected hamsters died, giving rise to fatality rates of 0%, 16.7%, and 83.3%, respectively (Fig. 1a). Progressive mean body weight loss of up to 93.9 ± 1.5%, 90.2 ± 4.2% and 82.6 ± 3.4% was observed from 0 to 5 dpi (Fig. 1b and Fig. S1a). Afterward, the body weights of Omicron BA.1-infected hamsters gradually recovered, while those for the prototype and Beta variant further dropped to 86.9 ± 5.7% and 76.7 ± 3.7% at 7 dpi, respectively, before they started to recover (Fig. 1b and Fig. S1a). In another parallel experiment, three hamsters were euthanized at 1, 3, 5, and 7 dpi. We then analyzed viral replication in respiratory tract organs, including the nasal turbinates, trachea and lungs, by real-time reverse transcription polymerase chain reaction (RT‒PCR) that amplified SARS-CoV-2 open reading frame 1ab (ORF1ab) for detection of viral RNA load in these homogenized tissues. The viral RNA levels in the turbinates of Omicron BA.1-infected hamsters were higher than those in the prototype-infected hamsters but lower than those in the Beta-infected hamsters at 1 dpi (Fig. 1c, left). The viral RNA levels in the turbinates of the prototype- and Beta-infected hamsters were maintained at over 1 × 10^7^ copies/mL from 3 to 7 dpi, whereas those of the Omicron BA.1-infected animals decreased to less than 1 × 10^5^ copies/mL at 7 dpi (Fig. 1c, left). Similar patterns were observed in the viral RNA levels in the tracheas of hamsters infected with these three strains (Fig. 1c, middle). Of note, from 1 to 7 dpi, the viral RNA levels in the lungs of Omicron BA.1-infected hamsters were significantly lower than those in the other two virus-infected groups (Fig. 1c, right). These results suggested that Omicron BA.1 had an attenuated replication ability in the respiratory tracts of adult hamsters. In addition, the Omicron BA.1-infected adult hamsters showed dose-dependent body weight loss attenuation and recovery (Fig. S2).

**Fig. 2:**
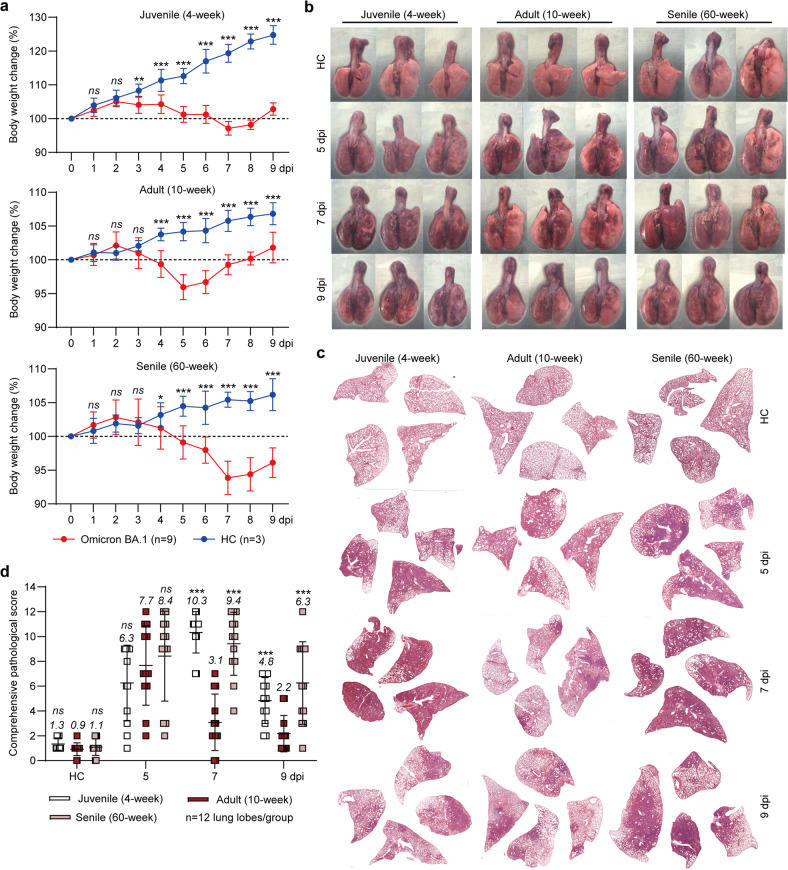
Pathogenicity of the SARS-CoV-2 Omicron BA.1 variant in juvenile, adult, and aged hamsters. **a** Body weight loss of healthy controls (HCs) (*n* = 3) and SARS-CoV-2-infected male hamsters (*n* = 9) from 0 to 9 dpi. In each age group, three hamsters were euthanized at 5, 7, and 9 dpi. The healthy controls were euthanized at 9 dpi. **b** Gross lung images, **c** representative H&E staining of lung lobes and **d** comprehensive pathological scores (for each group, 12 lung lobes collected from three hamsters were evaluated). We compared juvenile/aged hamsters to adult hamsters. Significance was calculated using two-way ANOVA (*P < 0.05; **P < 0.01; ***P < 0.001; ns nonsignificant)

As physiological states and immunological response patterns vary by age, which might affect the disease outcomes of Omicron BA.1 infection, we next compared the pathogenicity and replication efficiency of Omicron BA.1 in juvenile, adult, and aged hamsters. After intranasal inoculation with 1 × 10^5^ PFU of Omicron BA.1, the juvenile hamsters exhibited a significant arrest of body weight growth (Fig. 2a, upper and Fig. S1b); the adult hamsters showed 95.9 ± 1.9% body weight loss from 3 to 5 dpi and a rapid recovery from 6 to 9 dpi (Fig. 2a, middle and Fig. S1b); and the aged hamsters showed 93.7 ± 2.5% body weight loss from 3 to 7 dpi, with slow recovery (Fig. 2a, bottom and Fig. S1b). For each age group, three hamsters were euthanized at 5, 7, and 9 dpi. The gross lung tissue images showed moderate lung injury in adult hamsters at 5 dpi and severe lung injury in juvenile and aged hamsters at 5 and 7 dpi (Fig. 2b). Severe lung injury in juvenile hamsters largely recovered by 9 dpi, but recovery in the aged group was slow (Fig. 2b). The results from hematoxylin and eosin (H&E) staining of fixed lung lobes (Fig. 2c and Fig. S3) and comprehensive pathological scoring (Fig. 2d and Table S1) further confirmed that the Omicron BA.1-infected juvenile and aged hamsters had more severe and prolonged lung injury than the adult hamsters.

**Fig. 3:**
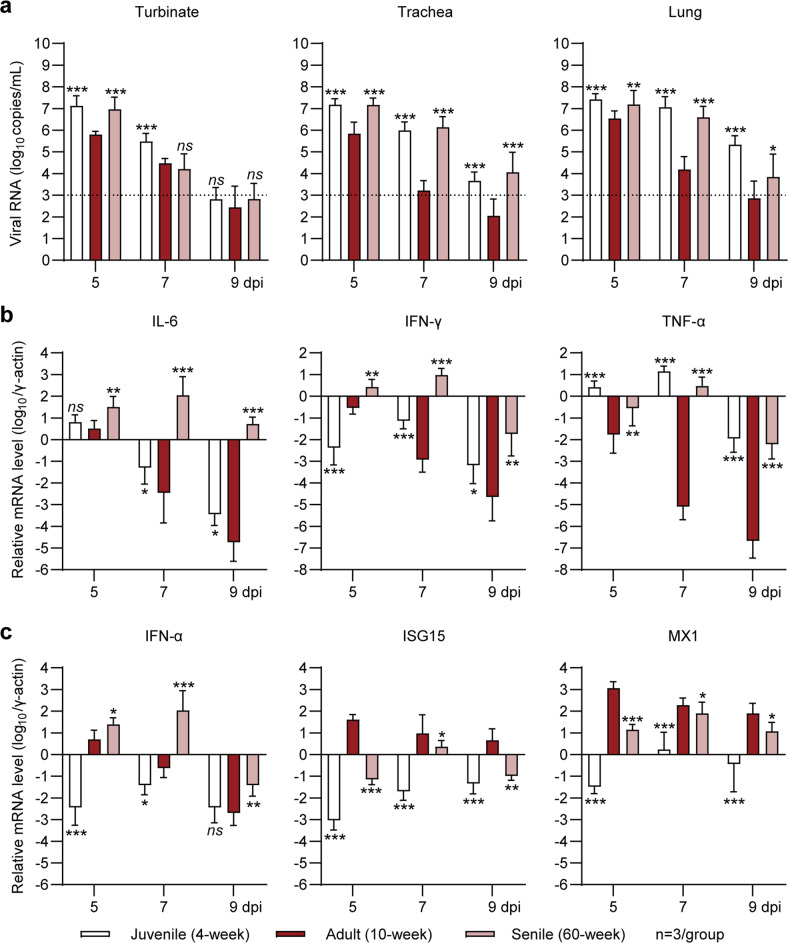
Measurement of viral RNA levels and the mRNA levels of proinflammatory cytokines and type I IFN-associated genes in respiratory tract organs of SARS-CoV-2 Omicron BA.1-infected juvenile, adult, and aged hamsters. **a** The viral RNA loads in respiratory tract organs, including the turbinates, trachea, and lungs, were measured by RT‒PCR at 5 dpi (*n* = 3). The fold changes in the mRNA levels of (**b**) proinflammatory cytokines and (**c**) IFN-α-associated genes in the lung tissues collected at 5 dpi (*n* = 3) are shown. The mRNA levels were standardized to those of the housekeeping gene γ-actin. We compared juvenile/aged hamsters to adult hamsters. Significance was calculated using two-way ANOVA (*P < 0.05; **P < 0.01; ***P < 0.001; ns nonsignificant)

To explore the viral burden in different age groups, we analyzed viral replication in respiratory tract organs, including the nasal turbinates, trachea, and lungs, by RT‒PCR amplification of SARS-CoV-2 ORF1ab for detection of the viral RNA load in homogenized tissues collected at 5, 7, and 9 dpi. The viral RNA levels in the turbinates were 10-fold higher in juvenile and aged hamsters than in adult hamsters at 5 dpi (Fig. 3a, left), while those in the trachea and lungs were 10- to 1000-fold higher in juvenile and aged hamsters than in adult hamsters from 5 to 9 dpi (Fig. 3a, middle and left). These results suggested a significant delay in viral clearance in Omicron BA.1-infected juvenile and aged hamsters. As viral clearance efficiency and the severity of pneumonia caused by SARS-CoV-2 are both closely related to host innate immune responses, we next measured the mRNA levels of typical proinflammatory cytokines and type I interferon (IFN)-related genes in homogenized lung tissues by RT‒PCR. The juvenile and aged hamsters showed 10- to 10000-fold higher expression of typical proinflammatory cytokines, including interleukin 6 (IL-6), interferon gamma (IFN-γ), and tumor necrosis factor alpha (TNF-α), than the adult hamsters at 7 and 9 dpi (Fig. 3b), revealing prolonged and exacerbated pulmonary inflammation. On the other hand, the juvenile hamsters showed 100- to 10000-fold lower expression of IFN-α and two critical interferon stimulated genes (ISGs), ISG15 and myxovirus resistance protein 1 (MX1), than the adult hamsters at 5 and 7 dpi (Fig. 3c), suggesting an inadequate type I IFN response. Interestingly, the aged hamsters showed 10- to 1000-fold higher expression of IFN-α than the adult hamsters from 5 and 9 dpi but 10- to 100-fold lower expression of ISG15 and MX1 (Fig. 3c), suggesting a skewing of the type I IFN response in Omicron BA.1-infected aged hamsters. Furthermore, we found that the IFN-γ concentration in serum was consistent with the IFN-γ mRNA levels in lung tissues (Fig. S4). In all three age groups, the serum IFN-γ concentration was positively related to the lung pathological score of the indicated hamster (Fig. S4). In conclusion, we demonstrated that adult hamsters developed an appropriate type I IFN response to achieve rapid clearance of Omicron BA.1 infection with transient and moderate pneumonia. However, the dysregulated innate immune responses in juvenile and aged hamsters impaired viral clearance and caused more severe and prolonged pneumonia.

**Fig. 4:**
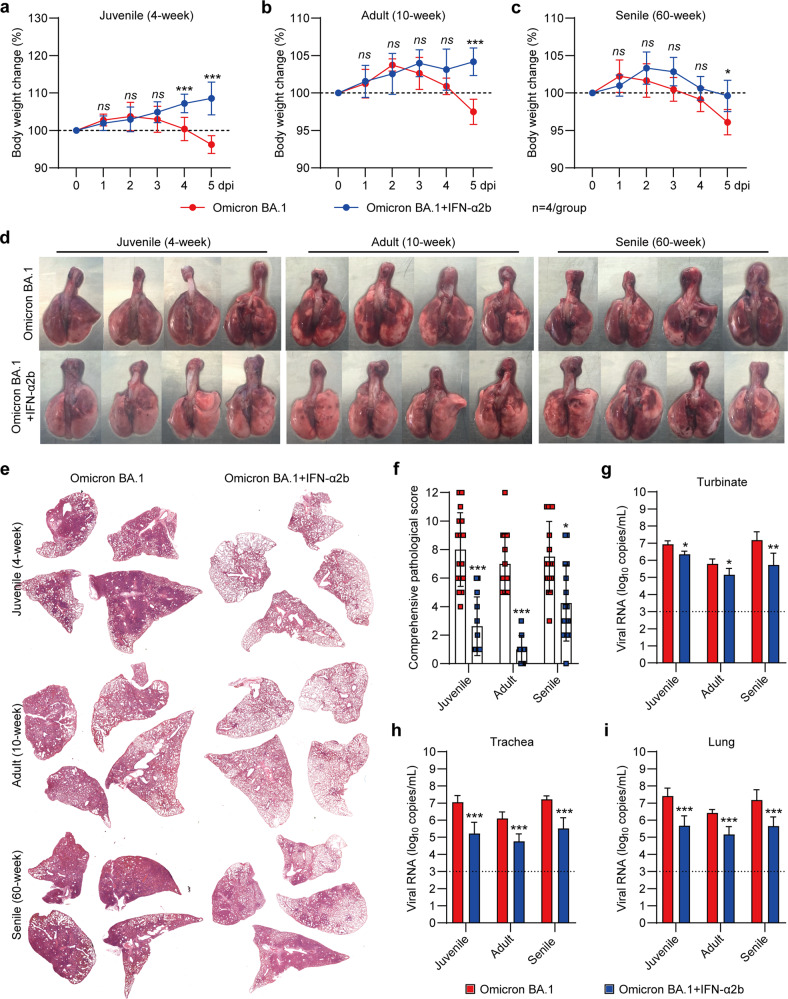
Early IFN-α2b treatment efficiently suppressed SARS-CoV-2 Omicron BA.1 replication and lung pathogenesis. Juvenile and aged male hamsters were intranasally inoculated with 1 × 10^5^ PFU of SARS-CoV-2 Omicron BA.1 and then administered three doses of recombinant IFN-α2b treatment at 24, 25, and 26 h post-infection. The body weight changes of SARS-CoV-2 Omicron BA.1-infected (**a**) juvenile, (**b**) adult and (**c**) aged hamsters and hamsters with early IFN-α2b treatment were measured from 0 to 5 dpi (*n* = 4/group). All hamsters were euthanized at 5 dpi. **d** Gross lung images, **e** representative H&E staining of lung lobes and **f** comprehensive pathological scores are shown (for each group, 16 lung lobes collected from four hamsters were evaluated). The viral RNA loads in respiratory tract organs, including the (**g**) turbinates, (**h**) trachea and (**i**) lungs, were measured by RT‒PCR (*n* = 4/group). We analyzed the differences between the hamsters with and without early IFN-α2b treatment in each age group. Significance was calculated using an unpaired two-tailed *t* test (*P < 0.05; **P < 0.01; ***P < 0.001; ns nonsignificant)

Based on the above findings, we hypothesized that early intervention with IFN-α might be helpful to improve the disease outcome of Omicron BA.1 infection in juvenile and adult hamsters. To validate this hypothesis, Omicron BA.1-infected juvenile and adult hamsters were intranasally administered three doses of recombinant IFN-α2b at 24, 25, and 26 h post-infection. The juvenile hamsters treated with IFN-α2b showed a 108.6 ± 4.4% body weight increase, whereas the untreated hamsters showed 96.2 ± 2.4% body weight loss at 5 dpi (Fig. 4a). The adult hamsters treated with IFN-α2b showed a 104.2 ± 1.8% body weight increase, whereas the untreated hamsters showed 97.5 ± 1.7% body weight loss at 5 dpi (Fig. 4b). The aged hamsters treated with IFN-α2b showed a 104.2 ± 1.8% body weight increase, whereas the untreated hamsters showed 97.5 ± 1.7% body weight loss at 5 dpi (Fig. 4c). For further virological and histological analysis, all of these juvenile, adult and aged hamsters were euthanized at 5 dpi. The results of gross lung imaging (Fig. 4d), H&E staining of fixed lung lobes (Fig. 4e and Fig. S5) and comprehensive pathological scoring (Fig. 4f and Table S2) demonstrated that juvenile and adult hamsters with early IFN-α2b treatment had significantly less lung injury than those without treatment. Furthermore, we analyzed viral replication in respiratory tract organs, including the turbinates, trachea, and lungs, by RT‒PCR amplification of SARS-CoV-2 ORF1ab for detection of the viral RNA load in the homogenized tissues collected at 5 dpi. The juvenile, adult, and aged hamsters with early IFN-α2b treatment showed 10- to 100-fold lower viral RNA levels in their turbinates, tracheas, and lung tissues than those without treatment (Fig. 4g–i), suggesting strong suppression of viral replication and accelerated viral clearance induced by IFN treatment.

## Discussion

The evolving SARS-CoV-2 mutates to adapt to its hosts, resulting in the emergence of new variant strains with varied disease outcomes after infection. Sensitive animal models can replicate the infectivity and pathogenicity of SARS-CoV-2 in human patients, providing valuable information on specific variant strains [20, 21]. After the emergence of Omicron, its virological characteristics, such as its varied hACE2 binding affinity compared with that of the Delta strain [22], insufficient usage of TMPRSS2 [23], immune escape and shifted tropism to upper respiratory tract tissues [24], were shown in cell models. However, whether these changes affect the disease outcome needs further validation in animal models.

As expected, the disease severity of Omicron BA.1 infection was shown to be milder than that of the SARS-CoV-2 prototype virus and Beta variant in adult hamsters, which parallels preliminary clinical data [6, 11] and the findings of previous animal studies [16, 17]. The attenuated replication efficiency and rapid viral clearance in the lungs are supposed to be two reasons for the low pathogenicity of Omicron BA.1 in adult hamsters. However, we further demonstrated that Omicron BA.1 could establish elevated replication and induce severe pneumonia in juvenile and aged hamsters. Compared with adult hamsters, both juvenile and aged hamsters exhibited prolonged increases in viral RNA levels in the respiratory tract. Omicron BA.1 infection largely impeded the normal body weight increase in the juvenile hamsters. The Omicron BA.1-infected aged hamsters showed prolonged body weight loss and slower recovery than the juvenile and adult hamsters. This might have been attributable to the different innate immune response patterns in the different age groups. The innate immune response mediated by type I IFN and its relevant functional signaling pathway is the crucial first step of host antiviral defense against acute SARS-CoV-2 infection [25–27]. In general, the adequate and appropriate innate immune response in adult hamsters cleared the virus, resulting in only transient inflammation and mild to moderate tissue injury. However, the innate immune response in juvenile hamsters was immature or insufficient to control the infection of Omicron BA.1 in time, resulting in prolonged viral replication and persistent viral attack. In contrast, an aging innate immune system may predispose the host to severe disease due to delayed and excessive release of proinflammatory cytokines [28], which has been demonstrated by a clinical study [29] and our results in aged hamsters infected with Omicron BA.1.

Age has been considered an important biological factor of the host response against infectious disease. The immune system changes with age in nearly every aspect, generally resulting in a decline in pathogen immunity and an increase in the proinflammatory response with increased age [28]. Single-cell analysis of peripheral blood mononuclear cells in young and aged adults has demonstrated that aging induces an immune dysfunction shift into effector and inflammatory cell populations [30]. For instance, natural killer cells and B cells lose their capacity for antiviral activity with upregulated inflammatory states in aging. Moreover, enhanced inflammatory signals and impaired regulatory signals between T cells and monocytes or natural killer cells and monocytes slow recovery in elderly patients. On the other hand, naïveté, abnormality and impairment in the immune system might interfere with or suppress the protective immune response after virus exposure or vaccination. Clinically, considering that the vaccine protection efficiency might be further reduced in populations with abnormal immune responses, such as newborn, juvenile, aged, pregnant and immunodeficient populations, Omicron may maintain its features as a highly pathogenic virus.

In the early stage of outbreaks, the threat of Omicron was underestimated, probably because of its milder pathogenicity and the herd immunity in a broad population that was conferred by prior infections and vaccinations. Although Omicron can largely escape low levels of neutralizing antibodies, ancestral SARS-CoV-2-specific T cells have been demonstrated to cross-recognize Omicron and suppress viral replication to some degree [31]. However, Omicron has recently achieved full pandemic status in all age groups worldwide, and the great increase in the number of infection cases has led to more hospitalizations and deaths, as seen during the pandemics of previous VOCs [32]. As most therapeutic antibodies are abolished or severely impaired by Omicron [8–10], host–target agents may become good options for urgent treatment of Omicron infection. In our study, early intervention via inhalation of IFN-α2b both suppressed viral replication and relieved lung pathogenesis in juvenile and adult hamsters, providing an accessible drug to reduce the increasing hospitalization rate. Regarding aged hamsters with an excessive innate immune response after Omicron infection, anti-inflammatory agents, such as dexamethasone, might be an optimal choice [33].

Since the emergence of BA.1, several new Omicron sublineages, including BA.2, BA.4, and BA.5, have become widespread worldwide. A recent study observed similar infectivity and pathogenicity in mice and hamsters for SARS-CoV-2 Omicron BA.1 and BA.2 [34]. Moreover, the current predominant SARS-CoV-2 Omicron sublineages BA.4 and BA.5 are more efficiently spread in human lung cells than BA.2. In addition, BA.4 and BA.5 are more pathogenic than BA.2 in hamsters [35]. Therefore, the manifestations and immune responses to BA.4/5 in juvenile and aged groups need more investigation in our future research.

**Fig. 5:**
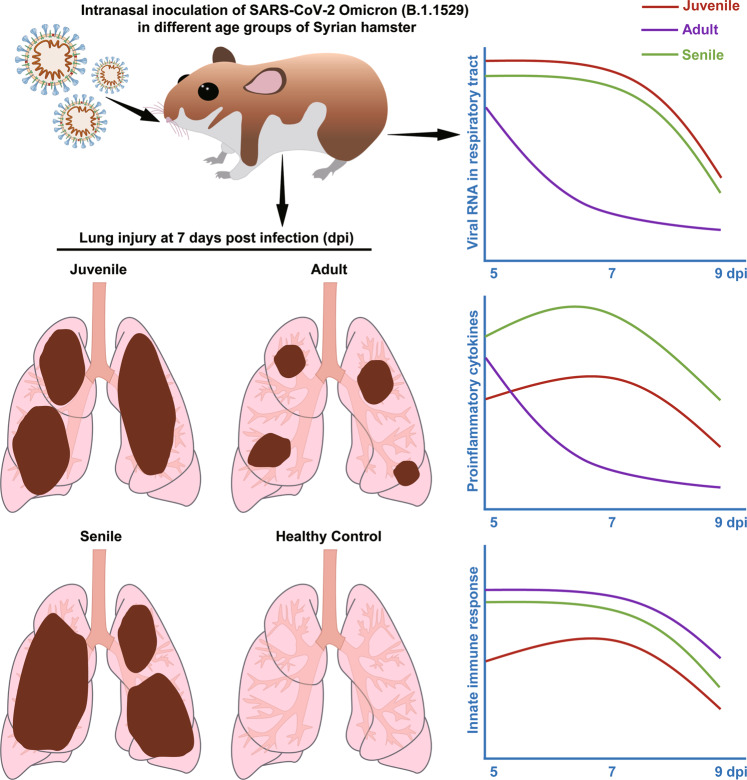
Schematic summary of SARS-CoV-2 Omicron infection and disease outcomes in juvenile, adult and aged hamsters. The Omicron variant of SARS-CoV-2 shows attenuated pathogenicity in adult hamsters compared with juvenile and aged hamsters, yet early interferon treatment can mitigate viral infection and improve disease outcomes

Taken together, our data confirm the attenuated disease outcomes of Omicron BA.1 infection in adult hamsters and demonstrate the elevated viral replication efficiency and pathogenicity of Omicron BA.1 in juvenile and aged hamsters (summarized in Fig. 5). These findings highlight the key role of the innate immune response in the progress of viral clearance and the regulation of pneumonia in different age groups. These critical findings upgrade our fundamental understanding of Omicron BA.1 infection and virus-host interplay and will improve the rational design/selection of prophylactic and therapeutic countermeasures to combat SARS-CoV-2 variants in the foreseeable future.

## Supplementary information

Supplementary Information Clean-copy
