# Supplementary material for: Infection, pathology and interferon treatment of the SARS-CoV-2 Omicron BA.1 variant in juvenile, adult and aged Syrian hamsters
Source: Cell Mol Immunol. 2022 Oct 18;19(12):1392–9. doi: 10.1038/s41423-022-00923-9 (PMC9579545; doi:10.1038/s41423-022-00923-9)
Supplement: Supplementary file 1 — Supplementary Information Clean-copy [file 41423_2022_923_MOESM1_ESM.docx]

**Supplementary Information**

**Infection, pathology and interferon treatment of SARS-CoV-2 Omicron variant in juvenile, adult and senile Syrian hamsters**

Lunzhi Yuan^1#^, Huachen Zhu^2,3,4#^, Peiwen Chen^2,3,4#^, Ming Zhou^1^, Jian Ma^1^, Xuan Liu^1^, Kun Wu^1^, Rirong Chen^2,3,4^, Qiwei Liu^2,3,4^, Huan Yu^2,3,4^, Lifeng Li^2,3,4^, Jia Wang^2,3,4^, Yali Zhang^1^, Shengxiang Ge^1^, Quan Yuan^1^, Qiyi Tang^5^, Tong Cheng^1^*, Yi Guan^2,3,4^*, Ningshao Xia^1^*

**Materials and Methods**

**Experimental Animal and Biosafety**

The Golden Syrian Hamster was raised in the specific pathogen free animal feeding facilities. All the animal experiments were approved by the Medical Ethics Committee (SUCM2021-112). All experiments with infectious SARS-CoV-2 were performed in the biosafety level 3 (BSL-3) and animal biosafety level 3 (ABSL-3) facilities. Our staff wear powered air-purifying respirators that filter the air, and disposable coveralls when they culture the virus and handle animals that are in isolators. The researchers are disinfected before they leave the room and then shower on exiting the facility. All facilities, procedures, training records, safety drills, and inventory records are subject to periodic inspections and ongoing oversight by the institutional biosafety officers who consult frequently with the facility managers.

**Virus Stock**

The SARS-CoV-2 prototype strain AP-8 (EPI_ISL_1655937), Beta variant strain AP-100 (EPI_ISL_2779638) and Omicron BA.1 variant strain (share an identical sequence with EPI_ISL_8182026) were passaged on Vero cells (#CCL-81, ATCC). Viral stocks were prepared in Vero cells with DMEM containing 2% FBS, 5ug/mL TPCK-trypsin, Penicillin-Streptomycin and 30mmol/L MgCl_2_ (#11995, #10270106, #T1426 and #15140-122; purchased from GIBCO, SIGMA-ALDRICH and Invitrogen). Viruses were harvested and stored in ultra-low temperature refrigerator. The titers were determined by means of plaque assay in Vero cells.

**Virus Inoculation and Sample Collection**

The hamsters were anesthetized by isoflurane (#R510-22, RWD Life Science) and the nasally inoculated with indicated doses of SARS-CoV-2 diluted in 200uL of PBS (#10010031, GIBCO). Body weight of these hamsters were measured by electronic balance. Hamsters were euthanized at indicated time point for detection of viral load in respiratory tract organs and analysis of pathogenesis in lung lobes.

**Administration of IFN-α2b**

The Omicron BA.1-infected hamsters received three doses of recombinant IFN-α2b (#202001E01, AmoTop) treatment at 24, 25 and 26 hours post infection, respectively.

For each dose, hamsters were treated with isoflurane lightly, after that, 2×10^6^ IU of recombinant IFN-α2b was dissolved in 200uL of PBS (#10010031, GIBCO) and administrated through intranasal route.

**Detection of Viral RNA**

Viral RNA was extracted by using a QIAamp Viral RNA Mini kit (#52906, Qiagen) according to the manufacturer's instructions. The RT-PCR was conducted by using the SLAN-96S Real-Time System (Hongshi, Shanghai, China) with a SARS-CoV-2 RT-PCR Kit from Wantai (Beijing, China). Relative Viral RNA of SARS-CoV-2 ORF1ab gene and NP gene were determined using primers pairs and probes shown in the kit instruction. Viral RNA copies were expressed on a log10 scale after normalized to the standard curve obtained by using ten-fold dilutions of a SARS-CoV-2 stock.

**Detection of Cytokine mRNA**

The lung tissues were cleaved into small pieces and soaked in RNAlater (#AM7021, Invitrogen). Total RNAs in lysed lung tissues were extracted with RNeasy Mini kit (#74106, Qiagen) and reverse-transcribed to cDNA with Fast-King Strand cDNA Synthesis Kit (#FP313, TIANGEN, Beijing) Diluted cDNAs (1:10) were quantified using SYBR Green I-based real-time PCR using the LightCycler® 480 instrument (Roche) per manufacturer’s instructions. Threshold cycle (Ct) of each gene was normalized to the internal reference gene (hamster γ-actin) and comparative Ct (2-ΔΔCt) method was utilized to calculate changes in chemokine and cytokine gene expression profile. The gene-specific primers (5’ to 3’) used for RT-PCR were listed in Supplementary Table S3.

**Detection for the concentration of IFN-γ in serum samples**

To measure the levels of IFN-γ in circulation system, serum samples collected from the SARS-CoV-2-infected juvenile, adult and senile hamsters were detected by a ELISA kit of hamster IFN-γ (#CSB-EL011050HA, CUSA Bio).

**Histopathological Studies**

For pathological analysis, lung tissues were fixed in formalin for more than 72 hours, dehydrated and then embedded in paraffin wax. The wax block of lung tissues was cut into 4μm sections for several pathological staining and analysis. H&E staining was employed for analysis of general lung pathogenic lesions include pulmonary edema, consolidation and inflammation. The standards for pathological score of lung tissues in this study are derived from our previous study in hamster model. Comprehensive pathological score of lung sections were performed according to the degree of lung lesions include alveolar septum hyperplasia, consolidation and impairment of alveolar structure, fluid exudation, mucus suppository, thrombus, inflammation recruitment and infiltration of immune cells in each individual lung lobes. For each hamster, three or four lung lobes were employed for evaluation of comprehensive pathological score. In brief, H&E staining result of each lung lobe was analyzed for its severity of pathological change. The pathological score include: a) Alveolar septum thickening and consolidation; b) Hemorrhage, exudation, pulmonary edema and mucous; c) Recruitment and infiltration of inflammatory immune cells. For each issue, score related to the severity: 0 indicate no pathological change was observed, 1 indicate moderate pathological change, 2 indicate mild pathological change, 3 indicate severe pathological change and 4 indicate very severe pathological change. In conclusion, scores of such three issues were added as the comprehensive pathological score of a lung lobe, and the average comprehensive pathological score of the lobes indicate the severity of lung pathogenesis in an evaluated hamster. The images of whole lung lobes were screened by a high-throughput screening microscope system (EVOS M7000, Invitrogen of Thermo Fisher Scientific).

**Statistical Analysis**

Data were presented as the means ± SD. Unpaired two-tailed t-test and two-way ANOVA were performed using GraphPad Prism 8.0 (GraphPad Software). p-values <0.05 were considered significant: *P <0.05, **P <0.01, ***P <0.001, ns indicates non-significant.


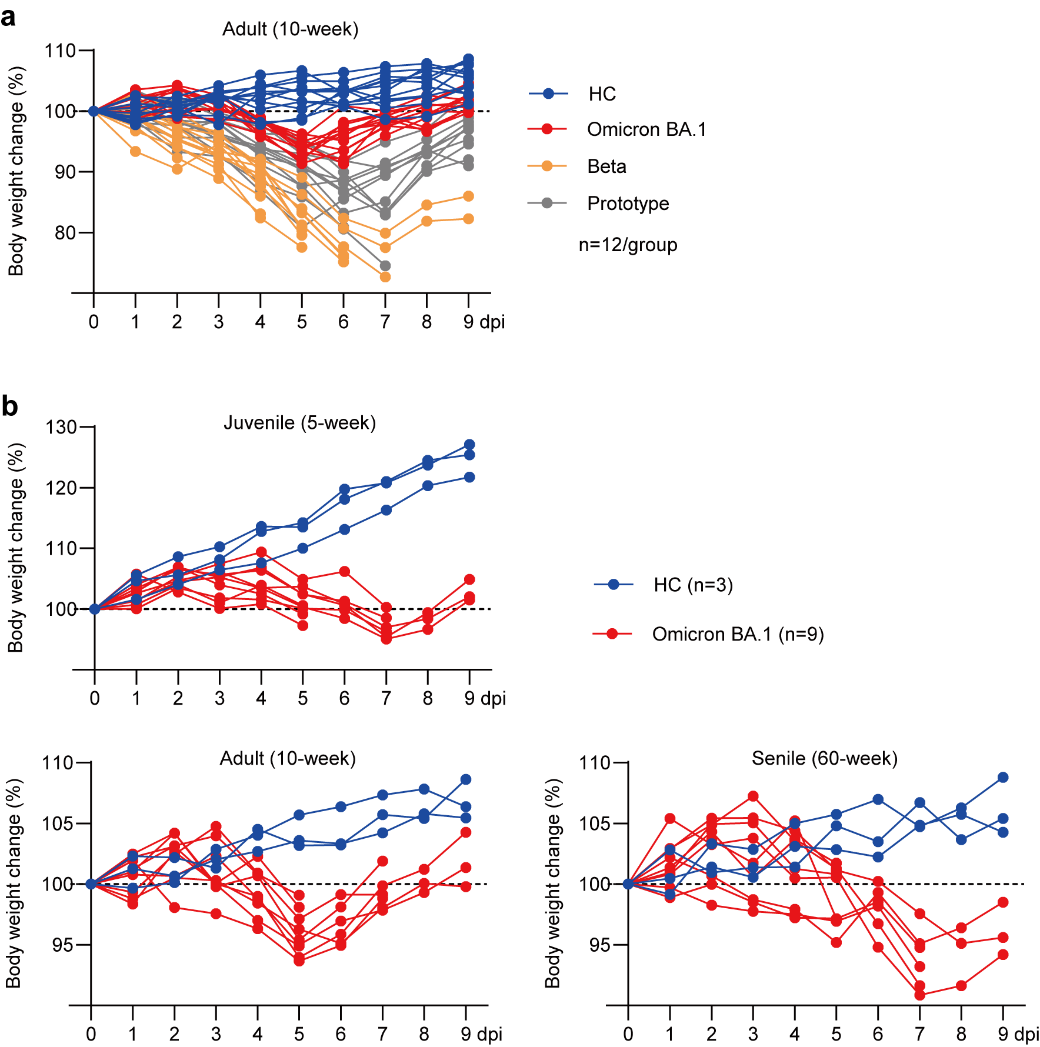


**figure S1.** (a) Bodyweight changes of individual healthy control (HC) and adult male hamsters intranasally inoculated with 1×10^5^ PFU of SARS-CoV-2 prototype, Beta and Omicron BA.1 variants, or PBS (healthy controls) from 0 to 9 dpi (n=12/groups). (b) Bodyweight changes of individual juvenile, adult and senile male hamsters intranasally infected with 1×10^5^ PFU of SARS-CoV-2 Omicron BA.1 (n=9), and the healthy controls (n=3) from 0 to 9 dpi.


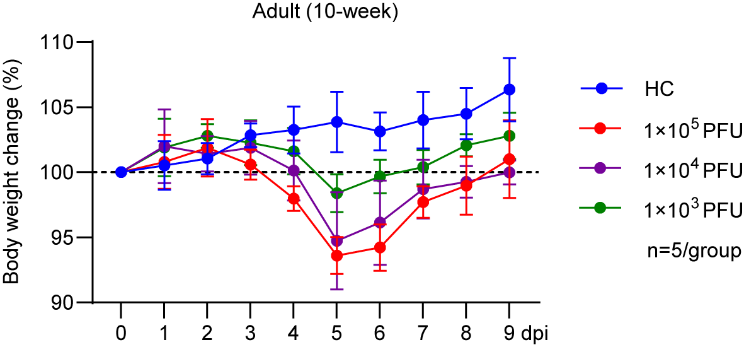


**figure S2.** Bodyweight changes of individual healthy control (HC) and adult male hamsters intranasally inoculated with different doses of SARS-CoV-2 Omicron BA.1 from 0 to 9 dpi (n=5/group).


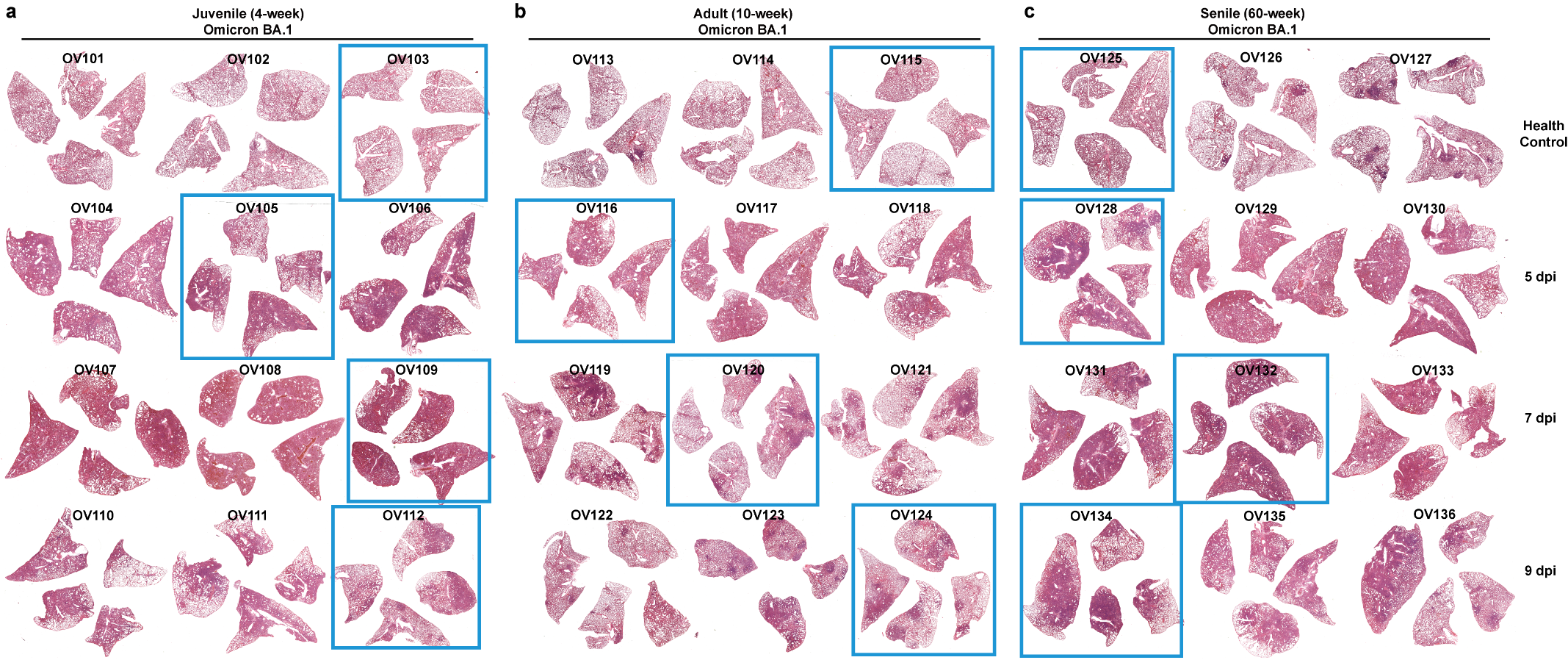


**figure S3.** H&E staining for the lung lobes collected from healthy controls and SARS-CoV-2 Omicron BA.1-infected hamsters euthanized at 5, 7 and 9 dpi. (a) H&E staining for lung lobes of SARS-CoV-2 Omicron BA.1-infected juvenile, (b) adult and (c) senile hamsters and healthy controls without infection. The images in blue frames were shown in Fig. 2c.


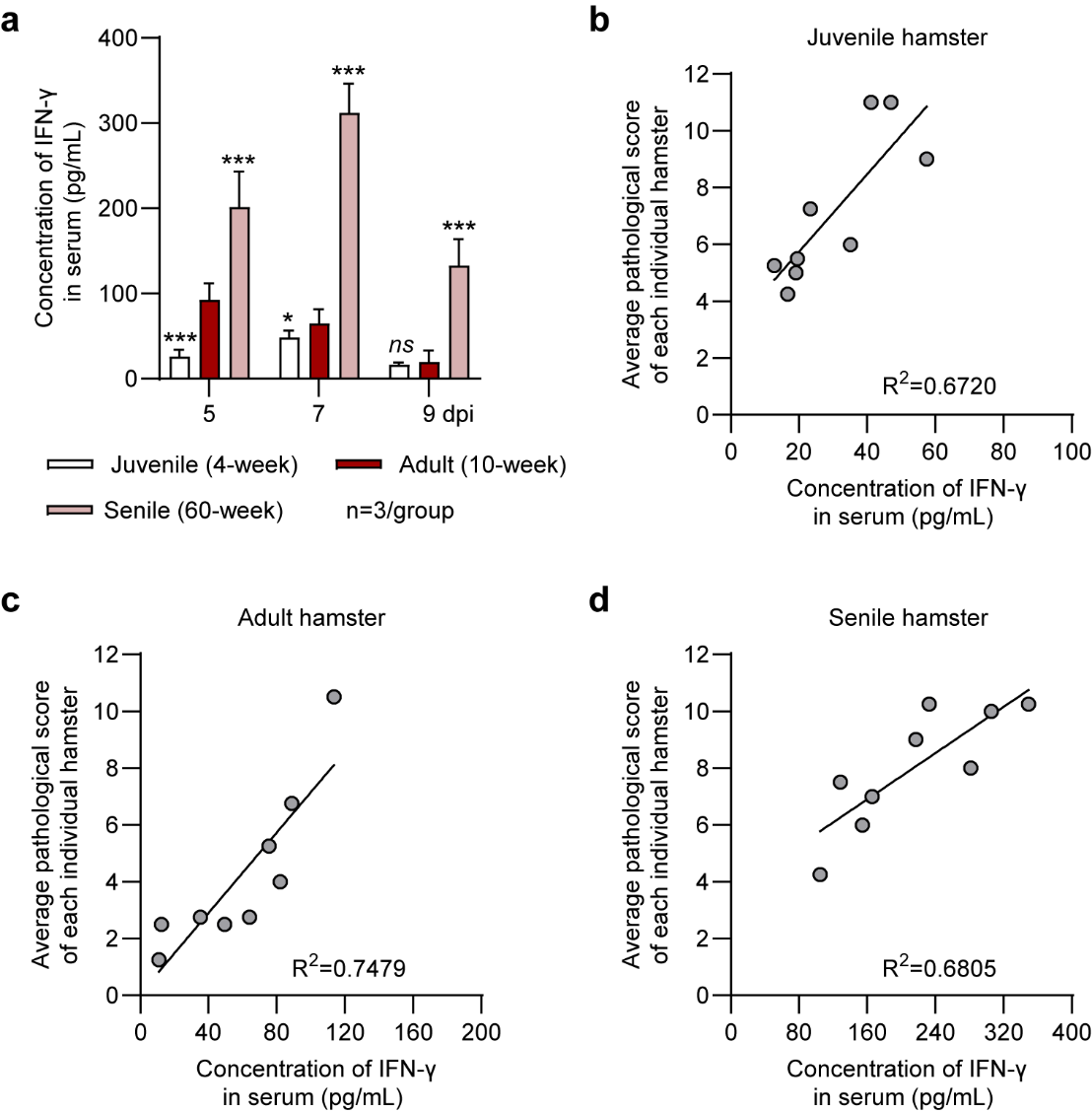


**figure S4.** Analysis for the concentration of IFN-γ in serum samples of SARS-CoV-2 BA.1-infected hamsters and their relationship between the severity of lung pathogenesis. (a) The concentration of IFN-γ in serum samples of SARS-CoV-2 BA.1-infected hamsters were measured by ELISA (n=3). We compared the juvenile/senile hamsters to the adult hamsters. Significance was calculated using two-way ANOVA (*P < 0.05; **P < 0.01; ***P < 0.001; ns, non-significant). The relationship between the concentration of IFN-γ in serum samples and the average pathological score for the lung lobes collected from indicated (b) juvenile, (c) adult and (d) senile hamsters were shown by linear regression analysis.


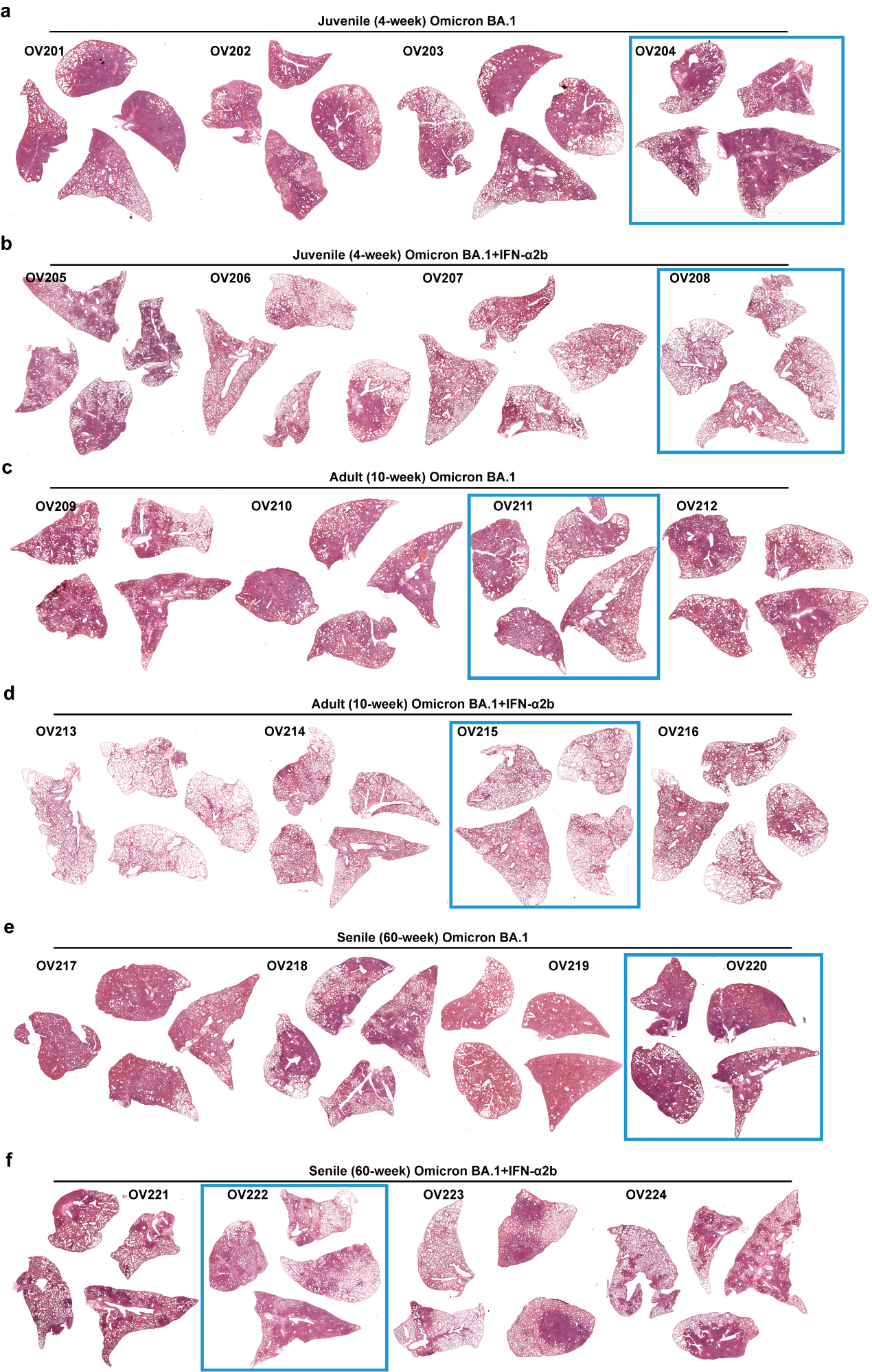


**figure S5.** H&E staining for the lung lobes collected from SARS-CoV-2 BA.1 infected hamsters with or without early inhaled interferon (IFN)-α2b treatment. All of the hamsters were euthanized at 5 dpi. H&E staining for lung lobes of SARS-CoV-2 Omicron BA.1 infected juvenile hamsters (b) with or (a) without IFN-α2b treatment. H&E staining for lung lobes of SARS-CoV-2 Omicron BA.1 infected adult hamsters (d) with or (c) without IFN-α2b treatment. H&E staining for lung lobes of SARS-CoV-2 Omicron BA.1 infected senile hamsters (f) with or (e) without IFN-α2b treatment. The images in blue frames were shown in Fig. 4d.

**Table S1. Comprehensive pathological scores for the H&E staining results of lung lobes in figure S3.**

| **Group** | | **Individual Identifier** | **Pathological lesions** | | | **Comprehensive pathological score** |  |
| --- | --- | --- | --- | --- | --- | --- | --- |
|  |  |  |  |  |  |  |  |
|  |  |  | **Alveolar septum hyperplasia and consolidation** | **Pulmonary edema, hemorrhage and mucus suppository** | **Recruitment and infiltration of inflammatory cells** |  |  |
|  |  |  |  |  |  |  |  |
| **Juvenile** | **HC** | **OV101** | 1+1+1+1 | 1+1+1+0 | 0+0+0+0 | 2+2+2+1 |  |
|  |  | **OV102** | 0+0+0+0 | 1+1+1+1 | 0+0+0+0 | 1+1+1+1 |  |
|  |  | **OV103** | 1+0+0+0 | 1+1+1+1 | 0+0+0+0 | 2+1+1+1 |  |
|  | **5 dpi** | **OV104** | 3+2+2+1 | 2+2+2+2 | 3+2+2+1 | 8+6+6+4 |  |
|  |  | **OV105** | 3+3+1+0 | 3+3+1+1 | 3+3+1+0 | 9+9+3+1 |  |
|  |  | **OV106** | 3+3+3+1 | 3+3+3+1 | 3+3+3+0 | 9+9+9+2 |  |
|  | **7 dpi** | **OV107** | 4+4+2+2 | 4+4+3+3 | 3+3+2+2 | 11+11+7+7 |  |
|  |  | **OV108** | 4+4+4+4 | 4+4+4+4 | 3+3+3+3 | 11+11+11+11 |  |
|  |  | **OV109** | 4+4+3+3 | 4+4+3+3 | 4+4+4+4 | 12+12+10+10 |  |
|  | **9 dpi** | **OV110** | 2+3+3+1 | 1+2+2+1 | 1+2+2+0 | 4+7+7+2 |  |
|  |  | **OV111** | 2+1+1+1 | 2+2+2+1 | 3+2+2+2 | 7+5+5+4 |  |
|  |  | **OV112** | 2+2+1+0 | 2+2+1+1 | 2+2+1+1 | 6+6+3+2 |  |
| **Adult** | **HC** | **OV113** | 0+0+0+0 | 1+1+1+1 | 1+0+0+0 | 2+1+1+1 |  |
|  |  | **OV114** | 0+0+0+0 | 1+1+1+0 | 0+0+0+0 | 1+1+1+0 |  |
|  |  | **OV115** | 0+0+0+0 | 1+1+1+0 | 0+0+0+0 | 1+1+1+0 |  |
|  | **5 dpi** | **OV116** | 2+2+2+1 | 2+2+2+1 | 3+3+2+1 | 7+7+6+3 |  |
|  |  | **OV117** | 4+4+3+3 | 4+4+4+4 | 3+3+3+3 | 11+11+10+10 |  |
|  |  | **OV118** | 4+2+2+0 | 4+2+2+1 | 4+3+2+1 | 12+7+6+2 |  |
|  | **7 dpi** | **OV119** | 2+1+1+0 | 2+2+1+1 | 3+1+1+0 | 7+4+3+2 |  |
|  |  | **OV120** | 2+0+0+0 | 2+1+1+0 | 2+1+1+0 | 6+2+2+0 |  |
|  |  | **OV121** | 2+1+1+0 | 2+1+1+0 | 2+1+0+0 | 6+3+2+0 |  |
|  | **9 dpi** | **OV122** | 1+0+0+0 | 1+1+1+1 | 0+0+0+0 | 2+1+1+1 |  |
|  |  | **OV123** | 2+0+0+0 | 1+1+1+1 | 3+1+1+1 | 5+2+2+2 |  |
|  |  | **OV124** | 1+1+0+0 | 2+1+0+0 | 2+1+1+1 | 5+3+1+1 |  |
| **Senile** | **HC** | **OV125** | 0+0+0+0 | 2+1+1+1 | 0+0+0+0 | 2+1+1+1 |  |
|  |  | **OV126** | 0+0+0+0 | 1+1+0+0 | 1+0+0+0 | 2+1+0+0 |  |
|  |  | **OV127** | 0+0+0+0 | 1+0+0+0 | 1+1+1+1 | 2+1+1+1 |  |
|  | **5 dpi** | **OV128** | 3+3+1+1 | 3+3+1+1 | 3+3+1+1 | 9+9+3+3 |  |
|  |  | **OV129** | 4+4+4+3 | 4+4+4+3 | 3+3+3+3 | 11+11+10+9 |  |
|  |  | **OV130** | 4+4+3+0 | 4+4+3+1 | 4+4+3+1 | 12+12+10+2 |  |
|  | **7 dpi** | **OV131** | 4+4+3+3 | 4+4+2+2 | 4+4+3+3 | 12+12+8+8 |  |
|  |  | **OV132** | 4+3+3+3 | 4+3+3+3 | 4+4+4+3 | 12+10+10+9 |  |
|  |  | **OV133** | 4+4+2+1 | 4+4+2+2 | 3+3+2+1 | 11+11+6+4 |  |
|  | **9 dpi** | **OV134** | 4+3+1+1 | 4+3+1+1 | 4+3+2+1 | 12+9+4+3 |  |
|  |  | **OV135** | 3+3+2+2 | 3+3+2+2 | 3+3+2+2 | 9+9+6+6 |  |
|  |  | **OV136** | 3+1+1+0 | 3+1+1+1 | 3+2+1+0 | 9+4+3+1 |  |

HC: healthy control; dpi: days post-inoculation/infection.

**Table S2. Comprehensive pathological scores for the H&E staining results of lung lobes in figure S5.**

| **Group** | | **Identifier** | **Pathological lesions** | | | **Comprehensive pathological score** |  |
| --- | --- | --- | --- | --- | --- | --- | --- |
|  |  |  |  |  |  |  |  |
|  |  |  | **Alveolar septum hyperplasia and consolidation** | **Pulmonary edema, hemorrhage and mucus suppository** | **Recruitment and infiltration of inflammatory cells** |  |  |
|  |  |  |  |  |  |  |  |
| **Juvenile** | **Omicron BA.1** | **OV201** | 4+3+3+1 | 4+3+3+2 | 4+4+4+2 | 12+10+10+5 |  |
|  |  | **OV202** | 3+2+2+2 | 3+2+2+2 | 3+3+2+2 | 9+7+6+6 |  |
|  |  | **OV203** | 4+3+3+2 | 4+3+3+2 | 4+3+3+1 | 12+9+9+5 |  |
|  |  | **OV204** | 3+2+2+1 | 4+2+2+2 | 4+3+2+1 | 11+7+6+4 |  |
|  | **Omicron BA.1+IFN-α2b** | **OV205** | 2+1+1+1 | 2+2+2+1 | 2+2+2+1 | 6+6+5+3 |  |
|  |  | **OV206** | 2+1+1+0 | 2+1+1+1 | 2+0+0+0 | 6+2+2+1 |  |
|  |  | **OV207** | 1+0+0+0 | 2+1+1+1 | 1+0+0+0 | 4+1+1+1 |  |
|  |  | **OV208** | 0+0+0+0 | 1+1+1+1 | 0+0+0+0 | 1+1+1+1 |  |
| **Adult** | **Omicron BA.1** | **OV209** | 2+2+2+1 | 3+2+2+2 | 3+2+2+2 | 8+6+6+5 |  |
|  |  | **OV210** | 4+3+2+2 | 4+3+2+2 | 4+3+2+2 | 12+9+6+6 |  |
|  |  | **OV211** | 3+3+1+1 | 3+2+2+2 | 3+2+2+2 | 9+7+5+5 |  |
|  |  | **OV212** | 3+3+1+1 | 3+3+2+2 | 3+3+2+2 | 9+9+5+5 |  |
|  | **Omicron BA.1+IFN-α2b** | **OV213** | 0+0+0+0 | 0+0+0+0 | 0+0+0+0 | 0+0+0+0 |  |
|  |  | **OV214** | 0+0+0+0 | 1+1+1+1 | 0+0+0+0 | 1+1+1+1 |  |
|  |  | **OV215** | 0+0+0+0 | 1+1+1+1 | 0+0+0+0 | 1+1+1+1 |  |
|  |  | **OV216** | 1+1+1+0 | 2+2+1+0 | 0+0+0+0 | 3+3+2+0 |  |
| **Senile** | **Omicron BA.1** | **OV217** | 3+3+3+2 | 2+2+2+1 | 3+2+2+2 | 8+7+7+5 |  |
|  |  | **OV218** | 2+2+1+1 | 2+2+2+1 | 2+2+2+1 | 6+6+5+3 |  |
|  |  | **OV219** | 4+4+2+2 | 4+4+3+3 | 3+3+2+2 | 11+11+7+7 |  |
|  |  | **OV220** | 4+4+3+2 | 3+3+3+2 | 4+4+3+2 | 11+11+9+6 |  |
|  | **Omicron BA.1+IFN-α2b** | **OV221** | 1+1+1+0 | 2+1+1+1 | 1+1+1+1 | 4+3+3+2 |  |
|  |  | **OV222** | 3+2+1+0 | 3+2+1+1 | 3+2+1+1 | 9+6+3+2 |  |
|  |  | **OV223** | 2+2+1+0 | 2+2+1+0 | 3+3+0+0 | 7+7+2+0 |  |
|  |  | **OV224** | 3+2+1+1 | 3+1+1+0 | 3+2+2+1 | 9+5+4+2 |  |

**Table S3. The gene-specific primers (5’ to 3’) used for RT-PCR of cytokine profiling**

| **Genes** | **Forward** | **Reverse** |
| --- | --- | --- |
| **IFN-γ** | TGTTGCTCTGCCTCACTCAGG | AAGACGAGGTCCCCTCCATTC |
| **IL-6** | AGACAAAGCCAGAGTCATT | TCGGTATGCTAAGGCACAG |
| **TNF-α** | TGAGCCATCGTGCCAATG | AGCCCGTCTGCTGGTATCAC |
| **γ-actin** | ACAGAGAGAAGATGACGCAGATAATG | GCCTGAATGGCCACGTACA |
| **IFN-α** | CTGGTGGCTGTGAGGAAATA | AGCAAGTTGGCTGAGGAAGA |
| **ISG15** | AAAGCCTACAGCCATGACCT | TTAGTCAGGGGCACCAGGAA |
| **MX1** | GCGCTTCCAGACTCTTCTGA | CCTAAGATACATGCGATGGCG |
